# Association of race, ethnicity and insurance status with outcomes for patients with acute pulmonary embolism treated by PERT: a retrospective observational study

**DOI:** 10.1186/s12931-024-02872-5

**Published:** 2024-06-24

**Authors:** Abdul Rehman, Avinash Singh, Priyanka Sridhar, Hong Yu Wang, Agostina Velo, Destiny Nguyen, Madeline Ehrlich, Robert Lookstein, David J. Steiger

**Affiliations:** 1 Department of Medicine, Rutgers-New Jersey Medical School, Newark, NJ 07103 USA; 2 Division of Pulmonary, Critical Care and Sleep Medicine, Department of Medicine, Icahn School of Medicine at Mount Sinai Health System, New York City, NY 10029 USA; 3 Department of Medicine, Icahn School of Medicine at Mount Sinai Health System, New York City, NY 10029 USA; 4 Department of Radiology, Icahn School of Medicine at Mount Sinai Health System, New York City, NY 10029 USA

**Keywords:** Pulmonary embolism, Venous thromboembolism, Racial groups, Ethnicity, Mortality, Length of stay

## Abstract

**Background:**

Management of PE has become streamlined with the implementation of PE Response Teams (PERT). Race, ethnicity and insurance status are known to influence the outcomes of patients with acute PE. However, whether the implementation of PERT-based care mitigates these racial and ethnic disparities remains unknown. Our aim was to assess the association of race, ethnicity and insurance with outcomes for patients with acute PE managed by PERT.

**Methods:**

We performed a retrospective chart review of 290 patients with acute PE, who were admitted to one of three urban teaching hospitals in the Mount Sinai Health System (New York, NY) from January 2021 to October 2023. A propensity score-weighted analysis was performed to explore the association of race, ethnicity and insurance status with overall outcomes.

**Results:**

Median age of included patients was 65.5 years and 149 (51.4%) were female. White, Black and Asian patients constituted 56.2% (163), 39.6% (115) and 3.5% [10] of the cohort respectively. Patients of Hispanic or Latino ethnicity accounted for 8.3% [24] of the sample. The 30-day rates of mortality, major bleeding and 30-day re-admission were 10.3%, 2.1% and 12.8% respectively. Black patients had higher odds of major bleeding (odds ratio [OR]: 1.445; *p* < 0.0001) when compared to White patients. Patients of Hispanic or Latino ethnicity had lower odds of receiving catheter-directed thrombolysis (OR: 0.966; *p* = 0.0003) and catheter-directed or surgical embolectomy (OR: 0.906; *p* < 0.0001) when compared to non-Hispanic/Latino patients. Uninsured patients had higher odds of receiving systemic thrombolysis (OR: 1.034; *p* = 0.0008) and catheter-directed thrombolysis (OR: 1.059; *p* < 0.0001), and lower odds of receiving catheter-directed or surgical embolectomy (OR: 0.956; *p* = 0.015) when compared to insured patients, although the odds of 30-day mortality and 30-day major bleeding were not significantly different.

**Conclusion:**

Within a cohort of PE patients managed by PERT, there were significant associations between race, ethnicity and overall outcomes. Hispanic or Latino ethnicity and uninsured status were associated with lower odds of receiving catheter-directed or surgical embolectomy. These results suggest that disparities related to ethnicity and insurance status persist despite PERT-based care of patients with acute PE.

**Supplementary Information:**

The online version contains supplementary material available at 10.1186/s12931-024-02872-5.

## Background

Worldwide, acute pulmonary embolism (PE) is the third leading cause of vascular disease after myocardial infarction and ischemic stroke [1]. The in-hospital mortality of PE averages around 6.7% but can be as high as 40% in patients with hemodynamically significant PE [2]. Almost a quarter of patients with acute PE may die before they reach the hospital [3]. In an effort to streamline the management of patients with acute PE, many institutions have created dedicated PE Response Teams (PERT) [4]. These teams comprise multidisciplinary experts in PE management that may include pulmonologists, cardiologists, cardiothoracic surgeons and interventional radiologists. A comprehensive plan of care is formulated by PERT for each acute PE in consultation with all members of the patient care team. Preliminary evidence suggests that the management of acute PE patients by PERT improves overall patient outcomes and reduces healthcare costs [5, 6]. 

PERT was implemented at our institution (Mount Sinai Health System in New York City, NY) beginning in 2018. The PERT consisted of the on-call pulmonologist, intensivist, cardiologist, cardiothoracic surgeon and interventional radiologist. The racial and ethnic makeup of the PERT was diverse. Clinicians included in the PERT were of different races including White, Black, Asian, Native American and Pacific Islander; a few clinicians of Hispanic or Latino ethnicity were also present. No community members were involved in the activities of PERT hitherto. Activation of a PERT consult was at the discretion of the primary attending (emergency physician, hospitalist or intensivist) taking care of the patient. The general criteria for activation of a PERT consult was the diagnosis of an acute PE, typically intermediate- or high-risk. In some cases, PERT consultation was also requested for patients with low-risk acute PE at the discretion of the treating physician. For each PERT consult, a management plan was formulated based on mutual discussion amongst all members of the PERT and the patient’s treating physician. The European Society of Cardiology 2019 guidelines [7] were used as the cornerstone of management for patients with acute PE as well as for risk stratification. Based on these guidelines, patient with hemodynamic instability were defined as high risk. Patients with both evidence of right heart strain (by echocardiography or computed tomography) and positive biomarkers (troponin and/or brain natriuretic peptide), but without any hemodynamic compromise, were defined as intermediate-high risk. Patients with either positive biomarkers or signs of right heart strain—but not both—in the absence of hemodynamic compromise—were defined as intermediate-low risk. Patients who were hemodynamically stable with normal biomarkers and no echocardiographic evidence of right heart strain were deemed low risk. The on-call interventional radiologist and the on-call cardiothoracic surgeon were available at all times of the day—and during all days of the week—to perform urgent or emergent reperfusion procedures as needed. Advanced reperfusion therapies offered to patients included catheter-directed thrombolysis (CDT), catheter-directed embolectomy and surgical embolectomy. For administering CDT, percutaneous central venous access was established and bilateral pulmonary artery catheters were placed. Alteplase bolus was administered through the catheters followed by a slow continuous infusion of alteplase over 16–24 h. Fibrinogen levels were used to titrate the rate of alteplase infusion. After completion of CDT, pulmonary angiography was performed to assess if CDT was successful. Catheter-directed embolectomy was performed using the FlowTriever® (Inari Medical, Irvine, CA) device—an over-the-wire mechanical thrombectomy device—after percutaneous central venous access. Surgical embolectomy was performed via a midline sternotomy and entailed direct removal of clots from the pulmonary trunk after total cardiopulmonary bypass. General indications for surgical embolectomy were failed CDT, failed catheter-directed embolectomy, hemodynamic instability precluding percutaneous intervention and radiographic signs of probable chronic thromboembolic pulmonary hypertension.

Race, ethnicity and insurance status are strong social determinants of health [8]. Racial and ethnic disparities are known to influence the outcomes of patients hospitalized with a diverse array of conditions [9–13]. Among patients hospitalized with acute PE, race and ethnicity have also been demonstrated to have an impact on overall outcomes [14–16]. However, whether the implementation of PERT-based care for patients with acute PE mitigates these racial and ethnic disparities remains unknown. Within the United States in general, and in the New York state in particular, people of color and those of Hispanic or Latino ethnicity have lower median income, literacy and health expectancy as compared to people of White race [17, 18]. These socioeconomic factors likely contribute to racial and ethnic disparities as well as potentially mediate such adverse outcomes.

In the present study, our aim was to assess the association of race, ethnicity and insurance status with overall outcomes for patients with acute PE managed by PERT. We hypothesized that the standardized management of acute PE patients through PERT would mitigate the impacts of racial group membership and insurance status on overall mortality and bleeding complications. We also explored the association of race and ethnicity with hospital length of stay (LOS) in this study.

## Methods

A retrospective observational cohort study was performed after obtaining approval from the institutional review board. Patients were treated in New York City (NY) at one of three urban hospitals in the Mount Sinai healthcare system viz. Mount Sinai Morningside, Mount Sinai Beth Israel and Mount Sinai West.

### Inclusion criteria

All patients diagnosed with acute PE and treated by PERT from January, 2021 to October, 2023 were eligible for inclusion in the study. PE was diagnosed by visualization of one or more filling defects within pulmonary arteries on contrast-enhanced computed tomography of the chest.

### Exclusion criteria

We excluded patients who had missing data on primary outcomes (hospital LOS, survival to discharge, and rates of 30-day mortality, 30-day bleeding and 30-day re-admission).

### Data collection

Eligible patients were identified by reviewing the list of PERT activations during the study period as well as by reviewing the records of patients who had an ICD-10-CM diagnosis code of pulmonary embolism. For all identified patients, the electronic health record was reviewed in a standardized and structured manner by study personnel, which entailed review of nursing observations, progress notes, radiology reports, laboratory investigations, insurance data, demographic information and biling records. This approach ensured that no bias was introduced as a consequence of differences in each personnel’s style of reviewing and extracting data from patients’ charts. The list of variables included included date of birth, date of admission, date of discharge, hospital LOS, disposition, location (within the hospital) of PE diagnosis, hospital-acquired PE, incidental PE, PESI score, ESC risk group, race, ethnicity, insurance, preferred language, sex, pleuritic chest pain, hemoptysis, dyspnea, syncope, altered mental status, unilateral leg pain or swelling, duration of symptoms, initial heart rate, initial temperature, initial systolic blood pressure, initial diastolic blood pressure, initial respiratory rate, initial pulse oximetry, oxygen support, body mass index (BMI), history of prior venous thromboembolism (VTE), presence of malignancy, immobility for 3 days, surgery less than 4 weeks prior, family history of thrombophilia, predisposing medications, recent long travel, active smoking, pregnancy, prior anticoagulation, history of cardiac disease, history of pulmonary hypertension, history of chronic lung disease, date and time of PE diagnosis, site of PE, RV strain by CT, RV strain by echocardiography, date and time of echocardiography, findings of echocardiography, lower extremity Doppler findings, date and time of lower extremity Doppler study, troponin I, brain natriuretic peptide, D-dimer, blood urea nitrogen, red cell distribution width (RDW) on initial presentation and discharge, creatinine, platelet count, international normalized ratio (INR), systemic anticoagulation, type of anticoagulant, inferior vena cava (IVC) filter, systemic thrombolysis, CDT, surgical or catheter-directed embolectomy, anticoagulant prescribed upon discharge, in-hospital bleeding and type of bleeding, survival to discharge, comfort care only, survival to 30 days, cause of death, 30-day bleeding and type of bleeding, 30-day re-admission, reason of re-admission, and follow-up with primary care physician, pulmonologist and hematologist. Membership in a particular racial or ethnic group was based on patients’ self-description. Primary and secondary insurance status were extracted directly from the electronic health record. The first set of vital signs documented in the medical chart by nursing staff were recorded as the “initial” vital signs, while laboratory investigations recorded were the first set on the day of presentation. Data was initially recorded on standardized spreadsheets and later imported into statistical software for final analysis.

The primary outcomes assessed for this study were hospital LOS, survival to discharge, and rates of 30-day mortality, 30-day bleeding and 30-day re-admission. Each mortality was discussed at a quarterly meeting of the PERT and the cause of death was determined after a thorough review and deliberation. PE was determined to be the cause of death if patient had evidence of worsening oxygenation, worsening perfusion or electromechanical dissociation prior to death. Major bleeding was defined as bleeding that resulted in a 2 g/dl drop in hemoglobin or required transfusion of 2 units packed red blood cells or any intracranial/intraspinal bleeding. Bleeding of any severity was defined as any report of subjective bleeding by the patient or objective documentation of bleeding by a healthcare professional—irrespective of the severity of bleeding or need for treatment. Follow-up was defined as an outpatient visit within 90 days of discharge. For this measure, we included both clinicians within the Mount Sinai Health System as well as out-of-network physicians.

### Statistical considerations

Statistical analysis was performed in R version 4.1.1. For all qualitative variables, frequencies were computed. For all quantitative variables, median and interquartile range (IQR) were computed. Propensity score weighting was performed using the *MatchIt* package for R. Propensity score was calculated from seven variables— age, sex, BMI, PESI score, ESC risk group, presence of a saddle PE and presence of malignancy—using a generalized linear model with the *probit* link function. Matching on the propensity score (propensity score-weighting) was performed using the optimal *full* matching specification, which does not place any constraints on the relative sizes of the control and treated groups [19, 20]. Density plots and standardized mean differences were examined to ensure the adequacy of balance within the matched dataset. Quasi-binomial regression models were used to assess the association of race (or ethnicity or insurance status) on in-hospital mortality, 30-day mortality, bleeding complications, utilization of advanced therapies and outpatient follow-up. Negative binomial regression model was used to assess the impact of race (or ethnicity or insurance status) on overall LOS. Odds ratios (OR) were computed from the quasi-binomial regression models, while incidence rate ratios (IRR) were computed from the negative binomial regression models. Cluster-robust standard errors were calculated using the *marginaleffects* package for R. In order to adjust for multiple comparisons, the Bonferroni correction method was applied to adopt a p-value cut-off of less than 0.0012 (= 0.05 ÷ 42) for statisticaly significance.

## Results

During the study period, 291 patients met inclusion criteria, out of which one patient was excluded due to missing data on primary outcomes. A total of 290 patients were included in the final analysis with a median age of 65.5 (IQR: 54.2‒76) years. There was a slight predominance of female patients (*n* = 149, 51.4%) in the study sample. White patients (*n* = 163, 56.2%) constituted the bulk of the sample followed by Black (*n* = 115, 39.6%) and Asian patients (*n* = 10, 3.5%). A small proportion of patients self-described as Hispanic or Latino (*n* = 24, 8.3%). Most patients included in the study were primarily English-speaking (*n* = 268, 92.4%), although a small proportion of patients required language interpreters (*n* = 22, 7.6%). Most patients had some form of medical insurance (*n* = 277, 95.5%) with the most common primary insurance being private insurance (*n* = 84, 29.0%) followed by government Medicare (*n* = 76, 26.2%) and commercial Medicare (*n* = 61, 21.0%). Government Medicaid and commercial Medicaid were the primary insurance for 21 (7.2%) and 35 (12.1%) patients respectively. The median BMI of included patients was 28.2 (IQR: 24.4‒34.6) kg/m^2^. A prior history of venous thromboembolism was reported in 60 (20.7%) patients and 11 (3.8%) patients were on anticoagulation prior to diagnosis of acute PE. The past medical history of study subjects included chronic lung disease (*n* = 46, 15.9%), congestive heart failure (*n* = 15, 5.2%) and pulmonary hypertension (*n* = 6, 2.1%). Risk factors for PE included active malignancy (*n* = 50, 17.2%), cigarette smoking (*n* = 31, 10.7%), immobility for three or more days (*n* = 23, 7.9%), surgery less than four weeks prior (*n* = 19, 6.5%), recent long flight (*n* = 17, 5.9%) and family history of thrombophilia (*n* = 12, 4.1%). Further details are provided in Table 1.

The most common clinical symptoms of acute PE were dyspnea (*n* = 207, 71.4%) and pleuritic chest pain (*n* = 106, 36.5%) followed by syncope (*n* = 60, 20.7%), altered mental status (*n* = 23, 7.9%) and hemoptysis (*n* = 10, 3.4%). The median duration of symptoms was 1 (IQR: 1‒3) day. Upon presentation, tachycardia, tachypnea, hypoxia and hypotension were noted in 88 (30.3%), 12 (4.1%), 20 (6.9%) and 16 (5.5%) patients respectively as detailed in Table 1. PE was incidentally diagnosed in 14 (4.8%) patients, while acute PE was hospital-acquired in 17 (5.9%) patients. Acute PE was the admitting diagnosis in most patients (*n* = 259, 89.3%) and was mostly diagnosed in the ED (*n* = 266, 91.7%). Medical or surgical intensive care unit (ICU) was the most common disposition for study patients (*n* = 260, 89.6%). The median PESI score for included patients was 82 (IQR: 63‒103) points. The ESC risk class for study subjects was low, intermediate-low, intermediate-high and high risk in 1.0% (*n* = 3), 20.7% (*n* = 60), 59.6% (*n* = 173) and 18.6% (*n* = 54) respectively. Central PE was diagnosed in 266 (91.7%) patients, while saddle PE was diagnosed in 58 (20.0%) patients. On CT scans, evidence of abnormal RV dilatation or straightening of interventricular septum was noted in 253 (87.2%) patients. Transthoracic echocardiography (TTE) was performed in 275 (94.8%) patients. Evidence of RV dysfunction was evident on TTE in 216 (74.5%) patients. The median troponin I and brain natriuretic peptide levels for our patients were 0.277 (IQR: 0.06‒2.05) ng/mL and 152.6 (IQR: 49.0‒339.3) pg/mL respectively. The median pulmonary artery systolic pressure on TTE was 39.6 (IQR: 30.8‒48.3) mm Hg. Lower extremity venous Doppler studies were performed in 221 (76.2%) patients and evidence of deep venous thrombosis was noted in 122 (42.1%) patients. The median D-dimer level for included patients was 10.42 (IQR: 4.97‒20.0) mg/L. Median platelet count, INR and RDW at the time of diagnosis of PE were 211 (IQR: 158‒262) × 10^9^ cells/L, 1.2 (IQR: 1.1‒1.3) and 13.1 (IQR: 12.2‒14.5) respectively. Moreover, the median levels of serum creatinine and blood urea nitrogen (BUN) were 0.95 (IQR: 0.76‒1.24) mg/dL and 17 (IQR: 12‒24) mg/dL respectively.

With respect to treatment, systemic anticoagulation was administered in nearly all patients (*n* = 286, 98.6%) with the initial choice of anticoagulation being unfractionated heparin and low molecular weight heparin in 77.6% (*n* = 225) and 20.3% (*n* = 59) of cases respectively. IVC filter was inserted in 40 (13.8%) patients. Systemic thrombolysis was administered in 13 (4.5%) patients, while CDT was administered in 16 (5.5%) patients. Surgical or catheter-directed embolectomy was performed in 37 (12.8%) patients.

**Table 1:** Demographic characteristics and clinical, laboratory & radiologic features of patients included in the study (*n* = 290)

Characteristics	Results
Age (median [IQR]), years	65.5 (54.2‒76)
*Sex*	
Female	149 (51.4%)
Male	141 (48.6%)
*Race*	
White	163 (56.2%)
Black	115 (39.6%)
Asian	10 (3.5%)
American Indian	1 (0.3%)
Other	1 (0.3%)
*Ethnicity*	
Hispanic or Latino	24 (8.3%)
Not Hispanic or Latino	266 (91.7%)
*Preferred language*	
English	268 (92.4%)
Spanish	18 (6.2%)
Chinese	3 (1.0%)
Albanian	1 (0.3%)
*Insurance status*	
Any insurance	277 (95.5%)
Uninsured	13 (4.5%)
*Primary insurance*	
Government Medicare	76 (26.2%)
Government Medicaid	21 (7.2%)
Commercial Medicare	61 (21.0%)
Commercial Medicaid	35 (12.1%)
Private insurance	84 (29.0%)
*Secondary insurance*	
Government Medicare	7 (2.4%)
Government Medicaid	48 (16.5%)
Commercial Medicare	1 (0.3%)
Commercial Medicaid	4 (1.4%)
Private insurance	26 (9.0%)
Body mass index, kg/m^2^	28.2 (24.3‒34.6)
*Past medical history*	
Prior episode of VTE	60 (20.7%)
On anticoagulation	11 (3.8%)
Congestive heart failure	15 (5.2%)
Chronic obstructive pulmonary disease	14 (4.8%)
Bronchial asthma	38 (13.1%)
Chronic lung disease	46 (15.9%)
Pulmonary hypertension	6 (2.1%)
*Risk factors for PE*	
Active malignancy	50 (17.2%)
Immobility for ≥ 3 days	23 (7.9%)
Surgery within 4 weeks	19 (6.5%)
Family history of thrombophilia	12 (4.1%)
Predisposing medications	11 (3.8%)
Recent long flight or travel	17 (5.9%)
Active smoker	31 (10.7%)
Pregnant	1 (0.3%)
*Symptoms*	
Pleuritic chest pain	106 (36.6%)
Hemoptysis	10 (3.5%)
Dyspnea	207 (71.4%)
Syncope	60 (20.7%)
Altered mental status	23 (7.9%)
Duration of symptoms (median [IQR]), days	1 [1–3]
*Clinical signs*	
Tachycardia	88 (%)
Low systolic blood pressure	16 (%)
Tachypnea	12 (%)
Hypoxia	20 (%)
PESI score (median [IQR])	82 (63‒103)
*ESC risk group*	
Low risk	3 (1.0%)
Intermediate-low risk	60 (20.7%)
Intermediate-high risk	173 (59.6%)
High risk	54 (18.6%)
*Site where PE diagnosed*	
Emergency department	266 (91.7%)
Inpatient	24 (8.3%)
*Disposition on admission*	
Intensive care unit	260 (89.6%)
Medical or surgical ward	30 (10.3%)
*Location of PE*	
Saddle (central)	58 (20.0%)
Main pulmonary artery (central)	129 (44.5%)
Lobar branch (central)	190 (65.5%)
Segmental branch (peripheral)	138 (47.6%)
Subsegmental (peripheral)	103 (35.5%)
*Associated CT findings*	
Pleural effusion	36 (12.4%)
Pulmonary infarct	42 (14.5%)
Abnormal RV function	253 (87.2%)
Echocardiography performed	275 (94.8%)
*Findings of echocardiography*	
PASP (median [IQR]), mm Hg	39.6 (30.9–48.3)
RV dilatation	196 (67.6%)
RV hypokinesia	198 (68.3%)
Venous Doppler study performed	221 (76.2%)
Evidence of DVT	122 (42.1%)
*Laboratory investigations (median [IQR])*	
Platelet count, 10^9^ cells/L	211 (158‒262)
RDW at the time of diagnosis of PE	13.1 (12.2‒14.5)
INR	1.2 (1.1‒1.3)
D-dimer, mg/L	10.4 (5.0‒20.0)
Troponin I, ng/mL	0.23 (0.06‒2.05)
Brain natriuretic peptide, pg/mL	153 (49‒339)

*CT =* Computed tomography; *DVT* = deep venous thrombosis; *ESC* = European Society of Cardiology; *IQR* = interquartile range; *LV* = left ventricle; *PASP* = pulmonary artery systolic pressure; *PE* = pulmonary embolism; *PESI* = PE Severity Index; *RDW* = red cell distribution width; *RV* = right ventricle; *VTE* = venous thromboembolism

In terms of overall outcomes, the in-hospital and 30-day mortality rates were 6.9% and 10.3% respectively (see Table 2). PE was deemed to be the cause of death in 8 (2.8%) cases. Among the patients who died in-hospital, comfort care only was pursued in 7 (2.4%) patients prior to death. The overall median hospital LOS for patients was 6 (IQR: 3‒10) days. In-hospital bleeding of any severity occurred in 23 (7.9%) patients, while in-hospital major bleeding occurred in only 5 (1.7%) patients. The most common type of anticoagulant prescribed upon discharge was apixaban (*n* = 169, 58.3%) followed by rivaroxaban (*n* = 29, 10.0%), enoxaparin (*n* = 20, 7.0%) and warfarin (*n* = 12, 4.1%). Of note, 32 (11.0%) patients were not prescribed any anticoagulant upon discharge, presumably due to risk of bleeding. The 30-day rate of bleeding of any severity was 9.0%, while the 30-day rate of major bleeding was only 2.1%. After discharge, 76.2% (*n* = 221), 25.9% (*n* = 75) and 15.9% (*n* = 46) of patients followed up in primary care, pulmonary and hematology clinics respectively. The 30-day rate of re-admission was 12.8% (*n* = 37). Bleeding and thrombotic complications accounted for 3 (1.0%) and 8 (2.8%) re-admissions respectively.

**Table 2:** Details of treatment and overall clinical outcomes of patients included in the study (*n* = 290)

Characteristics	Results
Systemic anticoagulation	286 (98.6%)
*Initial anticoagulant*	
Unfractionated heparin	225 (77.6%)
Low molecular weight heparin	59 (20.3%)
DOAC	2 (0.7%)
IVC filter insertion	40 (13.8%)
Systemic thrombolysis	13 (4.5%)
Catheter-directed thrombolysis	16 (5.5%)
Surgical or catheter-directed embolectomy	37 (12.8%)
*Anticoagulant prescribed upon discharge*	
Apixaban	169 (58.3%)
Dabigatran	6 (2.1%)
Rivaroxaban	29 (10.0%)
Warfarin	12 (4.1%)
Enoxaparin	20 (7.0%)
None	32 (11.0%)
*Overall outcomes*	
Length of stay (median [IQR]), days	6 [3–10]
In-hospital bleeding of any severity	23 (7.9%)
In-hospital major bleeding	5 (1.7%)
In-hospital death	20 (6.9%)
Death by thirty days	30 (10.3%)
Thirty-day bleeding of any severity	26 (9.0%)
Thirty-day major bleeding	6 (2.1%)
Thirty-day re-admission	37 (12.8%)
*Cause of in-hospital death*	
PE-related	8 (2.8%)
Other	12 (4.1%)
*Cause of re-admission*	
Thrombosis	8 (2.8%)
Bleeding	3 (1.0%)
Other	26 (9.0%)
*Outpatient follow-up*	
Primary care	221 (76.2%)
Pulmonary	75 (25.9%)
Hematology	46 (15.9%)

*DOAC* = Direct-acting oral anticoagulant; *PE* = pulmonary embolism; *IQR* = interquartile range; *IVC =* inferior vena cava

### Association of race with clinical outcomes

For the propensity score-weighted analysis, White patients were considered as the control (reference) group as they were the largest group by number (*n* = 163). In the first step, optimal full matching was performed to create a propensity score-weighted dataset whereby each Black patient was paired with one or more White patients. The density plot for this matched dataset is available in the online supplement for this article (Supplementary Fig. 1). The effective sample size for this propensity score-weighted dataset was 203.2. The results of regression analyses in the weighted dataset are detailed in Table 3. Patients of Black race had higher odds of 30-day major bleeding when compared to White patients (OR: 1.492; *p* < 0.0001). The hospital LOS and rates of 30-day mortality and 30-day re-admission were not significantly different for Black patients in comparison to White patients. The odds of following up in primary care, pulmonary or hematology clinics after discharge were not significantly different for Black patients when compared to White patients.

**Table 3:** Association of race with receipt of advanced therapies and overall outcomes

RACE	SYSTEMIC THROMBOLYSIS			
	Events	Odds ratio	95% CI	*p*-value
White (*n* = 163)	6	Reference	Reference	Reference
Black (*n* = 115)	7	1.021	0.967 to 1.079	0.454
Other (*n* = 12)	0	**0.862**	**0.800 to 0.930**	**< 0.0001**
**RACE**	**CATHETER-DIRECTED THROMBOLYSIS**			
	**Events**	**Odds ratio**	**95% CI**	***p*** **-value**
White (*n* = 163)	7	Reference	Reference	N/A
Black (*n* = 115)	7	1.029	0.986 to 1.074	0.192
Other (*n* = 12)	2	1.119	0.953 to 1.314	0.171
**RACE**	**SURGICAL or CATHETER-DIRECTED EMBOLECTOMY**			
	**Events**	**Odds ratio**	**95% CI**	***p*** **-value**
White (*n* = 163)	18	Reference	Reference	N/A
Black (*n* = 115)	17	1.043	0.970 to 1.121	0.255
Other (*n* = 12)	2	1.055	0.976 to 1.142	0.178
**RACE**	**IN-HOSPITAL MORTALITY**			
	**Events**	**Odds ratio**	**95% CI**	***p*** **-value**
White (*n* = 163)	12	Reference	Reference	N/A
Black (*n* = 115)	7	1.023	0.972 to 1.076	0373
Other (*n* = 12)	1	1.068	0.921 to 1.240	0.384
**RACE**	**MORTALITY AT THIRTY DAYS**			
	**Events**	**Odds ratio**	**95% CI**	***p*** **-value**
White (*n* = 163)	19	Reference	Reference	N/A
Black (*n* = 115)	9	0.982	0.925 to 1.043	0.565
Other (*n* = 12)	2	1.106	0.930 to 1.316	0.255
**RACE**	**IN-HOSPITAL BLEEDING OF ANY SEVERITY**			
	**Events**	**Odds ratio**	**95% CI**	***p*** **-value**
White (*n* = 163)	10	Reference	Reference	N/A
Black (*n* = 115)	12	1.054	0.994 to 1.117	0.076
Other (*n* = 12)	1	1.085	0.936 to 1.258	0.277
**RACE**	**IN-HOSPITAL MAJOR BLEEDING**			
	**Events**	**Odds ratio**	**95% CI**	***p*** **-value**
White (*n* = 163)	2	Reference	Reference	N/A
Black (*n* = 115)	3	**1.445**	**1.312 to 1.751**	**< 0.0001**
Other (*n* = 12)	0	**0.020**	**0.019 to 0.024**	**0.0001**
**RACE**	**BLEEDING OF ANY SEVERITY AT THIRTY DAYS**			
	**Events**	**Odds ratio**	**95% CI**	***p*** **-value**
White (*n* = 163)	12	Reference	Reference	N/A
Black (*n* = 115)	13	1.048	0.981 to 1.119	0.164
Other (*n* = 12)	1	1.084	0.935 to 1.256	0.284
**RACE**	**MAJOR BLEEDING AT THIRTY DAYS**			
	**Events**	**Odds ratio**	**95% CI**	***p*** **-value**
White (*n* = 163)	2	Reference	Reference	N/A
Black (*n* = 115)	4	**1.492**	**1.343 to 1.615**	**< 0.0001**
Other (*n* = 12)	0	**0.021**	**0.015 to 0.028**	**< 0.0001**
**RACE**	**LENGTH OF STAY (days)**			
	**Median (IQR)**	**IRR**	**95% CI**	***p*** **-value**
White (*n* = 163)	5 (2.25‒11.75)	Reference	Reference	N/A
Black (*n* = 115)	6 [3–10]	10.1	0.524 to 195.1	0.125
Other (*n* = 12)	4 (3‒6.75)	2.101	0.100 to 50.2	0.872
**RACE**	**THIRTY-DAY RE-ADMISSION**			
	**Events**	**Odds ratio**	**95% CI**	***p*** **-value**
White (*n* = 163)	20	Reference	Reference	N/A
Black (*n* = 115)	14	0.996	0.909 to 1.091	0.930
Other (*n* = 12)	3	**1.108**	**1.076 to 1.212**	**0.0002**
**RACE**	**PRIMARY CARE FOLLOW-UP**			
	**Events**	**Odds ratio**	**95% CI**	***p*** **-value**
White (*n* = 163)	119	Reference	Reference	N/A
Black (*n* = 115)	93	1.035	0.942 to 1.138	0.473
Other (*n* = 12)	9	1.119	0.985 to 1.269	0.084
**RACE**	**PULMONARY FOLLOW-UP**			
	**Events**	**Odds ratio**	**95% CI**	***p*** **-value**
White (*n* = 163)	45	Reference	Reference	N/A
Black (*n* = 115)	26	0.930	0.832 to 1.039	0.198
Other (*n* = 12)	4	0.989	0.763 to 1.281	0.932
**RACE**	**HEMATOLOGY FOLLOW-UP**			
	**Events**	**Odds ratio**	**95% CI**	***p*** **-value**
White (*n* = 163)	28	Reference	Reference	N/A
Black (*n* = 115)	15	0.964	0.878 to 1.060	0.446
Other (*n* = 12)	3	**1.165**	**1.093 to 1.271**	**0.0001**

* *p*-values and odds ratios computed using multivariate quasi-binomial regression models within the propensity score-weighted sample

*CI* = confidence interval; *IQR* = interquartile range; *IRR* = incidence rate ratio; *N/A* = not applicable; *OR* = odds ratio

Given the scarcity of patients of races other than White (*n* = 163) and Black (*n* = 115), they were all grouped together as a single “Other” race (*n* = 12). In the second step, optimal full matching was performed to create a propensity score-weighted dataset whereby each “Other” race patient was paired with one or more White patients. The density plot for this matched dataset is available in the online supplement for this article (Supplementary Fig. 2). The effective sample size for this propensity score-weighted dataset was 55.3. The results of regression analyses in the weighted dataset are detailed in Table 3. Patients of “Other” race had lower odds of receiving systemic thrombolysis when compared to White patients (OR: 0.862; *p* < 0.0001), although the odds of 30-day mortality were not significantly different. Moreover, patients of “Other” race had lower odds of 30-day major bleeding when compared to White patients (OR: 0.021; *p* < 0.0001). The hospital LOS was not significantly different for patients of “Other” race when compared to White patients. However, the odds of 30-day re-admission were slightly higher for patients of “Other” race when compared to White patients (OR: 1.108; *p* = 0.0002). Odds of following up in hematology clinics was slightly higher for patients of “Other” race when compared to White patients (OR: 1.165; *p* = 0.0001), although the odds of following up in primary care or pulmonary clinics were not significantly different.

### Association of ethnicity with clinical outcomes

For this analysis, patients of non-Hispanic or Latino ethnicity (*n* = 266) were considered as the control (reference group) and each patient of Hispanic or Latino ethnicity was paired with one or more patients in the control group using the optimal full matching specification. The density plot for this matched dataset is available in the online supplement for this article (Supplementary Fig. 3). The effective sample size for this propensity score-weighted dataset was 114.1. The results of regression analyses in the weighted dataset are detailed in Table 4. Patients of Hispanic or Latino ethnicity had lower odds of receiving CDT (OR: 0.966; *p* = 0.0003) and surgical or catheter-directed embolectomy (OR: 0.906; *p* < 0.0001) when compared to patients of non-Hispanic/Latino ethnicity. However, odds of in-hospital and 30-day mortality were not significantly different for Hispanic or Latino patients when compared to patients of other ethnicities. Interestingly, patients of Hispanic or Latino ethnicity had lower LOS when compared to patients of non-Hispanic or Latino ethnicity (IRR: 0.019; *p* = 0.0004). On the other hand, patients of Hispanic or Latino ethnicity also had lower odds of following up in pulmonary clinic when compared to patients of non-Hispanic/Latino ethnicity (OR: 0.852; *p* = 0.0009), although the odds of following up in primary care or hematology clinics were not significantly different.

**Table 4:** Association of ethnicity with receipt of advanced therapies and overall outcomes

ETHNICITY	SYSTEMIC THROMBOLYSIS			
	Events	Odds ratio	95% CI	*p*-value
Not Hispanic or Latino (*n* = 266)	13	Reference	Reference	Reference
Hispanic or Latino (*n* = 24)	0	0.968	0.930 to 0.989	0.0019
**ETHNICITY**	**CATHETER-DIRECTED THROMBOLYSIS**			
	**Events**	**Odds ratio**	**95% CI**	***p*** **-value**
Not Hispanic or Latino (*n* = 266)	16	Reference	Reference	N/A
Hispanic or Latino (*n* = 24)	0	**0.966**	**0.944 to 0.988**	**0.0003**
**ETHNICITY**	**SURGICAL or CATHETER-DIRECTED EMBOLECTOMY**			
	**Events**	**Odds ratio**	**95% CI**	***p*** **-value**
Not Hispanic or Latino (*n* = 266)	36	Reference	Reference	N/A
Hispanic or Latino (*n* = 24)	1	**0.906**	**0.858 to 0.957**	**< 0.0001**
**ETHNICITY**	**IN-HOSPITAL MORTALITY**			
	**Events**	**Odds ratio**	**95% CI**	***p*** **-value**
Not Hispanic or Latino (*n* = 266)	19	Reference	Reference	N/A
Hispanic or Latino (*n* = 24)	1	0.929	0.865 to 0.999	0.046
**ETHNICITY**	**MORTALITY AT THIRTY DAYS**			
	**Events**	**Odds ratio**	**95% CI**	***p*** **-value**
Not Hispanic or Latino (*n* = 266)	26	Reference	Reference	N/A
Hispanic or Latino (*n* = 24)	4	1.012	0.935 to 1.104	0.764
**ETHNICITY**	**IN-HOSPITAL BLEEDING OF ANY SEVERITY**			
	**Events**	**Odds ratio**	**95% CI**	***p*** **-value**
Not Hispanic or Latino (*n* = 266)	23	Reference	Reference	N/A
Hispanic or Latino (*n* = 24)	0	**0.878**	**0.837 to 0.921**	**< 0.0001**
**ETHNICITY**	**IN-HOSPITAL MAJOR BLEEDING**			
	**Events**	**Odds ratio**	**95% CI**	***p*** **-value**
Not Hispanic or Latino (*n* = 266)	5	Reference	Reference	N/A
Hispanic or Latino (*n* = 24)	0	0.977	0.950 to 0.997	0.015
**ETHNICITY**	**BLEEDING OF ANY SEVERITY AT THIRTY DAYS**			
	**Events**	**Odds ratio**	**95% CI**	***p*** **-value**
Not Hispanic or Latino (*n* = 266)	26	Reference	Reference	N/A
Hispanic or Latino (*n* = 24)	0	**0.867**	**0.833 to 0.903**	**< 0.0001**
**ETHNICITY**	**MAJOR BLEEDING AT THIRTY DAYS**			
	**Events**	**Odds ratio**	**95% CI**	***p*** **-value**
Not Hispanic or Latino (*n* = 266)	6	Reference	Reference	N/A
Hispanic or Latino (*n* = 24)	0	0.980	0.955 to 0.998	0.035
**ETHNICITY**	**LENGTH OF STAY (days)**			
	**Median (IQR)**	**IRR**	**95% CI**	***p*** **-value**
Not Hispanic or Latino (*n* = 266)	6.0 (3.0‒11.0)	Reference	Reference	N/A
Hispanic or Latino (*n* = 24)	4.5 (2.0‒8.0)	**0.019**	**0.009 to 0.189**	**0.0004**
**ETHNICITY**	**THIRTY-DAY RE-ADMISSION**			
	**Events**	**Odds ratio**	**95% CI**	***p*** **-value**
Not Hispanic or Latino (*n* = 266)	32	Reference	Reference	N/A
Hispanic or Latino (*n* = 24)	5	1.152	1.008 to 1.317	0.038
**ETHNICITY**	**PRIMARY CARE FOLLOW-UP**			
	**Events**	**Odds ratio**	**95% CI**	***p*** **-value**
Not Hispanic or Latino (*n* = 266)	203	Reference	Reference	N/A
Hispanic or Latino (*n* = 24)	18	0.964	0.881 to 1.055	0.422
**ETHNICITY**	**PULMONARY FOLLOW-UP**			
	**Events**	**Odds ratio**	**95% CI**	***p*** **-value**
Not Hispanic or Latino (*n* = 266)	73	Reference	Reference	N/A
Hispanic or Latino (*n* = 24)	2	**0.852**	**0.770 to 0.943**	**0.0009**
**ETHNICITY**	**HEMATOLOGY FOLLOW-UP**			
	**Events**	**Odds ratio**	**95% CI**	***p*** **-value**
Not Hispanic or Latino (*n* = 266)	42	Reference	Reference	N/A
Hispanic or Latino (*n* = 24)	4	1.011	0.913 to 1.119	0.829

* *p*-values and odds ratios computed using multivariate quasi-binomial regression models within the propensity score-weighted sample; incidence rate ratios computed using multivariate negative binomial regression models within the propensity score-weighted sample

*CI* = confidence interval; *IQR* = interquartile range; *IRR* = incidence rate ratio; *N/A* = not applicable; *OR* = odds ratio

### Association of insurance status with clinical outcomes

For this analysis, patients with any insurance (*n* = 277) were considered as the control (reference) group and each uninsured patient (*n* = 13) was paired with one or more patients in the control group using the optimal full matching specification. The density plot for this matched dataset is available in the online supplement for this article (Supplementary Fig. 4). The effective sample size for this propensity score-weighted dataset was 59.6. The results of regression analyses in the weighted dataset are detailed in Table 5. Uninsured patients had lower odds of receiving systemic thrombolysis (OR: 1.034; *p* = 0.0008), CDT (OR: 1.059; *p* < 0.0001) and surgical or catheter-directed embolectomy (OR: 0.906; *p* < 0.0001) when compared to insured patients. However, the odds of 30-day mortality (OR: 1.050; *p* = 0.176), 30-day major bleeding (OR: 0.999; *p* = 0.518) and 30-day re-admission (OR: 1.128; *p* = 0.636) for uninsured patients were not significantly different than those of insured patients. Hospital LOS for uninsured patients was also not significantly different from that of insured patients (IRR: 3.069 [95% CI: 0.104‒90.3]; *p* = 0.516). Likewise, the odds of following up in primary care (OR: 0.975; *p* = 0.525), pulmonary (OR: 1.029; *p* = 0.450) or hematology (OR: 1.043; *p* = 0.480) clinics for uninsured patients were not significantly different from those of insured patients.

**Table 5:** Association of insurance status with receipt of advanced therapies and overall outcomes

INSURANCE STATUS	SYSTEMIC THROMBOLYSIS			
	Events	Odds ratio	95% CI	*p*-value
Insured (*n* = 277)	12	Reference	Reference	Reference
Uninsured (*n* = 13)	1	**1.034**	**1.009 to 1.060**	**0.0008**
**INSURANCE STATUS**	**CATHETER-DIRECTED THROMBOLYSIS**			
	**Events**	**Odds ratio**	**95% CI**	***p*** **-value**
Insured (*n* = 277)	15	Reference	Reference	N/A
Uninsured (*n* = 13)	1	**1.059**	**1.042 to 1.077**	**< 0.0001**
**INSURANCE STATUS**	**SURGICAL or CATHETER-DIRECTED EMBOLECTOMY**			
	**Events**	**Odds ratio**	**95% CI**	***p*** **-value**
Insured (*n* = 277)	36	Reference	Reference	N/A
Uninsured (*n* = 13)	1	**0.906**	**0.898 to 0.951**	**< 0.0001**
**INSURANCE STATUS**	**IN-HOSPITAL MORTALITY**			
	**Events**	**Odds ratio**	**95% CI**	***p*** **-value**
Insured (*n* = 277)	18	Reference	Reference	N/A
Uninsured (*n* = 13)	2	1.065	0.979 to 1.158	0.142
**INSURANCE STATUS**	**MORTALITY AT THIRTY DAYS**			
	**Events**	**Odds ratio**	**95% CI**	***p*** **-value**
Insured (*n* = 277)	28	Reference	Reference	N/A
Uninsured (*n* = 13)	2	1.050	0.978 to 1.127	0.176
**INSURANCE STATUS**	**IN-HOSPITAL BLEEDING OF ANY SEVERITY**			
	**Events**	**Odds ratio**	**95% CI**	***p*** **-value**
Insured (*n* = 277)	22	Reference	Reference	N/A
Uninsured (*n* = 13)	1	0.971	0.884 to 1.067	0.546
**INSURANCE STATUS**	**IN-HOSPITAL MAJOR BLEEDING**			
	**Events**	**Odds ratio**	**95% CI**	***p*** **-value**
Insured (*n* = 277)	5	Reference	Reference	N/A
Uninsured (*n* = 13)	0	1.000	0.998 to 1.001	0.654
**INSURANCE STATUS**	**BLEEDING OF ANY SEVERITY AT THIRTY DAYS**			
	**Events**	**Odds ratio**	**95% CI**	***p*** **-value**
Insured (*n* = 277)	25	Reference	Reference	N/A
Uninsured (*n* = 13)	1	0.954	0.854 to 1.066	0.404
**INSURANCE STATUS**	**MAJOR BLEEDING AT THIRTY DAYS**			
	**Events**	**Odds ratio**	**95% CI**	***p*** **-value**
Insured (*n* = 277)	6	Reference	Reference	N/A
Uninsured (*n* = 13)	0	0.999	0.996 to 1.002	0.518
**INSURANCE STATUS**	**LENGTH OF STAY (days)**			
	**Median (IQR)**	**IRR**	**95% CI**	***p*** **-value**
Insured (*n* = 277)	6 (3‒9.25)	Reference	Reference	N/A
Uninsured (*n* = 13)	8 [6–17]	3.069	0.104 to 90.3	0.516
**INSURANCE STATUS**	**THIRTY-DAY RE-ADMISSION**			
	**Events**	**Odds ratio**	**95% CI**	***p*** **-value**
Insured (*n* = 277)	34	Reference	Reference	N/A
Uninsured (*n* = 13)	3	1.128	0.975 to 1.378	0.636
**INSURANCE STATUS**	**PRIMARY CARE FOLLOW-UP**			
	**Events**	**Odds ratio**	**95% CI**	***p*** **-value**
Insured (*n* = 277)	211	Reference	Reference	N/A
Uninsured (*n* = 13)	10	0.975	0.902 to 1.054	0.525
**INSURANCE STATUS**	**PULMONARY FOLLOW-UP**			
	**Events**	**Odds ratio**	**95% CI**	***p*** **-value**
Insured (*n* = 277)	73	Reference	Reference	N/A
Uninsured (*n* = 13)	2	1.029	0.955 to 1.108	0.450
**INSURANCE STATUS**	**HEMATOLOGY FOLLOW-UP**			
	**Events**	**Odds ratio**	**95% CI**	***p*** **-value**
Insured (*n* = 277)	44	Reference	Reference	N/A
Uninsured (*n* = 13)	2	1.043	0.928 to 1.172	0.480

* *p*-values and odds ratios computed using multivariate quasi-binomial regression models within the propensity score-weighted sample; incidence rate ratios computed using multivariate negative binomial regression models within the propensity score-weighted sample

*CI* = confidence interval; *IQR* = interquartile range; *IRR* = incidence rate ratio; *N/A* = not applicable; *OR* = odds ratio

### Association of preferred language with clinical outcomes and interaction with race, ethnicity and insurance status

To assess if patients’ preferred language had a confounding effect on the associations between clinical outcomes and race, ethnicity and insurance status, we applied Fisher’s exact tests to see if the proportion of patients preferring English differed based on race, ethnicity and insurance status. The results of these tests are detailed in Table 6. The proportion of patients who preferred English were significantly different based on racial group membership (*p* < 0.0001) and ethnicity (*p* < 0.0001).

**Table 6:** Association of preferred language with race, ethnicity and insurance status (*n* = 290)

GROUP		PREFERRED LANGUAGE		*p*-value*
		English	Other	
**RACE**	White (*n* = 163)	146	17	**< 0.0001**
	Black (*n* = 115)	114	1	
	Other (*n* = 12)	8	4	
**ETHNICITY**	Hispanic or Latino (*n* = 24)	11	13	**< 0.0001**
	Non-Hispanic/Latino (*n* = 266)	257	9	
**INSURANCE**	Insured (*n* = 277)	256	21	0.999
	Uninsured (*n* = 13)	12	1	

* *p-*value calculated using Fisher’s exact test

To further explore the association of preferred language with clinical outcomes, we performed another propensity score-weighted analysis. Patients who preferred English (*n* = 268) were considered as the control (reference) group and patients who preferred a language other than English (*n* = 22) were paired with one or more patients in the control group using the optimal full matching specification. The density plot for this matched dataset is available in the online supplement for this article (Supplementary Fig. 5). The effective sample size for this propensity score-weighted dataset was 90.6. The results of regression analyses in the weighted dataset are detailed in Table 7. Patients who preferred a language other than English had higher odds of receiving surgical or catheter-directed embolectomy (OR: 1.099; *p* < 0.0001) and had higher odds of 30-day bleeding of any severity (OR: 0.900; *p* < 0.0001) when compared to other patients. Moreover, the odds of in-hospital mortality were lower for patients who preferred a language other than English (OR: 0.944; *p* = 0.0003) as compared to other patients, although the odds of 30-day mortality and 30-day major bleeding were not significantly different. Moreover, hospital LOS, odds of 30-day re-admission and odds of following up in primary care, pulmonary and hematology clinics after discharge were not significantly different for patients who preferred a language other than English.

**Table 7:** Association of preferred language with receipt of advanced therapies and overall outcomes

PREFERRED LANGUAGE	SYSTEMIC THROMBOLYSIS			
	Events	Odds ratio	95% CI	*p*-value
English (*n* = 268)	13	Reference	Reference	Reference
Other (*n* = 22)	0	0.982	0.967 to 0.998	0.026
**PREFERRED LANGUAGE**	**CATHETER-DIRECTED THROMBOLYSIS**			
	**Events**	**Odds ratio**	**95% CI**	***p*** **-value**
English (*n* = 268)	15	Reference	Reference	N/A
Other (*n* = 22)	1	1.013	0.996 to 1.029	0.129
**PREFERRED LANGUAGE**	**SURGICAL or CATHETER-DIRECTED EMBOLECTOMY**			
	**Events**	**Odds ratio**	**95% CI**	***p*** **-value**
English (*n* = 268)	33	Reference	Reference	N/A
Other (*n* = 22)	4	**1.099**	**1.060 to 1.140**	**< 0.0001**
**PREFERRED LANGUAGE**	**IN-HOSPITAL MORTALITY**			
	**Events**	**Odds ratio**	**95% CI**	***p*** **-value**
English (*n* = 268)	20	Reference	Reference	N/A
Other (*n* = 22)	0	**0.944**	**0.915 to 0.974**	**0.0003**
**PREFERRED LANGUAGE**	**MORTALITY AT THIRTY DAYS**			
	**Events**	**Odds ratio**	**95% CI**	***p*** **-value**
English (*n* = 268)	31	Reference	Reference	N/A
Other (*n* = 22)	4	1.075	0.950 to 1.216	0.252
**PREFERRED LANGUAGE**	**IN-HOSPITAL BLEEDING OF ANY SEVERITY**			
	**Events**	**Odds ratio**	**95% CI**	***p*** **-value**
English (*n* = 268)	23	Reference	Reference	N/A
Other (*n* = 22)	0	**0.910**	**0.889 to 0.932**	**< 0.0001**
**PREFERRED LANGUAGE**	**IN-HOSPITAL MAJOR BLEEDING**			
	**Events**	**Odds ratio**	**95% CI**	***p*** **-value**
English (*n* = 268)	5	Reference	Reference	N/A
Other (*n* = 22)	0	0.997	0.991 to 1.003	0.302
**PREFERRED LANGUAGE**	**BLEEDING OF ANY SEVERITY AT THIRTY DAYS**			
	**Events**	**Odds ratio**	**95% CI**	***p*** **-value**
English (*n* = 268)	26	Reference	Reference	N/A
Other (*n* = 22)	0	**0.900**	**0.873 to 0.928**	**< 0.0001**
**PREFERRED LANGUAGE**	**MAJOR BLEEDING AT THIRTY DAYS**			
	**Events**	**Odds ratio**	**95% CI**	***p*** **-value**
English (*n* = 268)	6	Reference	Reference	N/A
Other (*n* = 22)	0	0.986	0.967 to 1.005	0.158
**PREFERRED LANGUAGE**	**LENGTH OF STAY (days)**			
	**Median (IQR)**	**IRR**	**95% CI**	***p*** **-value**
English (*n* = 268)	6.0 (3.6‒10.1)	Reference	Reference	N/A
Other (*n* = 22)	8.5 (4.5‒13.6)	0.334	0.030 to 3.709	0.372
**PREFERRED LANGUAGE**	**THIRTY-DAY RE-ADMISSION**			
	**Events**	**Odds ratio**	**95% CI**	***p*** **-value**
English (*n* = 268)	33	Reference	Reference	N/A
Other (*n* = 22)	4	1.070	1.007 to 1.137	0.029
**PREFERRED LANGUAGE**	**PRIMARY CARE FOLLOW-UP**			
	**Events**	**Odds ratio**	**95% CI**	***p*** **-value**
English (*n* = 268)	203	Reference	Reference	N/A
Other (*n* = 22)	18	1.031	0.959 to 1.108	0.405
**PREFERRED LANGUAGE**	**PULMONARY FOLLOW-UP**			
	**Events**	**Odds ratio**	**95% CI**	***p*** **-value**
English (*n* = 268)	73	Reference	Reference	N/A
Other (*n* = 22)	2	0.854	0.772 to 0.945	0.002
**PREFERRED LANGUAGE**	**HEMATOLOGY FOLLOW-UP**			
	**Events**	**Odds ratio**	**95% CI**	***p*** **-value**
English (*n* = 268)	45	Reference	Reference	N/A
Other (*n* = 22)	1	0.905	0.832 to 0.984	0.019

* *p*-values and odds ratios computed using multivariate quasi-binomial regression models within the propensity score-weighted sample; incidence rate ratios computed using multivariate negative binomial regression models within the propensity score-weighted sample

*CI* = confidence interval; *IQR* = interquartile range; *IRR* = incidence rate ratio; *N/A* = not applicable; *OR* = odds ratio

Patients of Hispanic or Latino ethnicity had lower odds of receiving surgical or catheter-directed embolectomy, while patients who preferred a language other than English had higher odds of receiving surgical or catheter-directed embolectomy as compared to respective control groups. This suggests that the association between ethnicity and receipt of surgical or catheter-directed embolectomy was likely not confounded by preferred language. Moreover, there were no significant associations between preferred language and 30-day mortality, 30-day major bleeding or hospital LOS. This also suggests that the association of race and ethnicity with 30-day mortality, 30-day major bleeding or hospital LOS were not confounded by preferred language.

## Discussion

The results of our study demonstrated that the odds of 30-day mortality, 30 day re-admission and hospital LOS among Black patients diagnosed with acute PE—that was managed by PERT—were not significantly different when compared to those of White patients, although the odds of 30-day major bleeding for Black patients were higher. Moreover, the odds of mortality among patients of Asian and other races were not significantly different when compared to those of White patients. These results suggest that PERT-based care of acute PE patients possibly mitigated racial disparities in patient outcomes. Conversely, patients of Hispanic or Latino ethnicity had lower odds of receiving CDT and catheter-directed or surgical embolectomy when compared to non-Hispanic/Latino patients. These results suggest that PERT-based care of acute PE patients did not mitigate ethnic disparities in patient treatment. Uninsured patients had lower odds of receiving systemic thrombolysis, CDT and catheter-directed or surgical embolectomy when compared to insured patients, although odds of 30-day mortality and 30-day major bleeding were not significantly different.

Our study cohort consisted exclusively of patients who were treated for acute PE by PERT within three large urban teaching hospitals in New York City. Our patient population was diverse with respect to ethnic and racial makeup as well as health literacy and insurance status. Moreover, the PERT itself comprised of clinicians of different races and ethnicities. The 30-day mortality of our cohort was 10.3%, while the rates of 30-day major bleeding and 30-day re-admission were 2.1% and 12.8% respectively. These results were largely concordant with the outcomes reported by other investigators for acute PE in the era of PERT-based care. Within the National PERT Consortium, Schultz and colleagues observed an overall 30-day mortality rate of 16% and 30-day rate of major bleeding of 13% [21]. In another study, Rosovsky et al. investigated the outcomes of 228 patients with acute PE who were cared for by PERT and observed 30-day mortality and 30-day major bleeding rates of 5.1% and 5.7% respectively [22]. Moreover, Hussein et al. reported the outcomes of 819 patients with acute PE who were cared for by PERT and found that the median hospital LOS was 5 days [23]. These results were similar to those of our cohort where the median hospital LOS was 6 days. Overall, these results suggest that the quality of PERT-based care offered to patients in our cohort was largely similar to that offered by PERT at other institutions.

Race and ethnicity are strongly associated with overall outcomes of patients hospitalized with acute PE [14, 24]. In the Nationwide Inpatient Sample (NIS), Farmakis and colleagues demonstrated that patients of races other than White had higher odds of in-hospital mortality and lower odds of receiving advanced therapies for PE [14]. On the other hand, in our cohort of patients with acute PE managed by PERT, the odds of mortality for patients of races other than White were not significantly different than that of White patients. Our results were similar to those of Dronamraju et al., who observed that among 425 patients with acute PE managed by PERT, in-hospital treatment and in-hospital mortality were not significantly different between Black and White patients [25]. These results suggest that PERT-based care of acute PE patients can mitigate racial disparities in treatment and overall outcomes by providing standardized care to each patient.

In our study, patients of Hispanic or Latino ethnicity had lower odds of receiving CDT and catheter-directed or surgical embolectomy when compared to non-Hispanic/Latino patients. Moreover, patients of Hispanic or Latino ethnicity also had lower odds of following up in pulmonary clinic when compared to patients of non-Hispanic/Latino ethnicity. These results were similar to the findings of Sathianathan and colleagues, who observed that in the NIS, Hispanic patients were less likely to undergo CDT [24]. It is interesting to note that even though PERT-based care mitigated racial disparities in patient care in our cohort, ethnic disparities still persisted. One possible explanation may be that even for patients cared for by PERT, treatment plans that are formulated by different clinicians may not be completely standardized. Schultz and colleagues investigated the outcomes of different patients cared for by PERT within the National PERT Consortium and found that treatments delivered for acute PE and overall mortality varied greatly between PERTs at different institutions [21]. 

A major reason for the observed lack of a standardized management approach to PERT-based care for patients with acute PE is because the optimal therapy for high risk and intermediate risk PE remains incompletely defined. There is a paucity of randomized controlled studies comparing the efficacy and safety of different advanced therapies [26, 27]. Studies comparing different treatment regimens are mostly retrospective and non-randomized [28–30]. Most studies describing the utility of advanced therapies are single-arm studies [31–34]. In the absence of randomized controlled studies, there are retrospective cohort studies, systematic reviews and meta-analyses comparing different advanced therapies versus standard-of-care full dose anticoagulation mainly based on retrospective data [35]. There are consensus statements offering guidelines [36, 37] and there are ongoing randomized trials of CDT and catheter-directed embolectomy [38]. The HI-PEITHO study is randomizing intermediate high-risk PE patients to ultrasound-assisted CDT and anticoagulation versus full-dose anticoagulation [39]. Results of these on-going randomized trials may help to standardize the management of intermediate high-risk PE.

Race and ethnicity are known to be strong determinants of overall health [9–13, 40]. Recent research into race and ethnicity has underscored the arbitrariness of dividing humans into rigid groups based on a few phenotypic traits, such as hair and skin color [8]. Race is not a valid biological construct as traditionally defined racial and ethnic groups are not accurate reflections of genetically distinct populations [41]. Moreover, there is significant educational, economic and cultural heterogeneity within traditionally defined racial and ethnic groups [42]. All these research findings dictate that racial and ethnic differences in patient outcomes are largely driven by implicit biases, poor health literacy, differences in healthcare availability, underinsurance, economic disparities and social structures [8, 43]. In the context of the present study, we observed that the odds of 30-day mortality among patients of different racial groups were not significantly different, presumably due to standardized care offered by PERT to patients with acute PE, which mitigated racial disparities. At the same time, patients of Hispanic or Latino ethnicity had lower odds of receiving CDT and surgical or catheter-directed embolectomy, which reflects a persistence of ethnic disparities. These ethnic disparities could be mitigated as well by attending to patient, healthcare provider, and systemic factors.

Our patient cohort included a small number of patients of Asian, Pacific Islander and Native American races, which precluded a meaningful subgroup analysis of these patients. Nevertheless, we grouped all these patients together as a single “Other” race and explored their outcomes in propensity score-weighted analyses. Patients of “Other” race had lower odds of receiving systemic thrombolysis, although their odds of receiving CDT or catheter-directed/surgical embolectomy were not significantly different. Moreover, patients of “Other” race had lower odds of major bleeding at 30-days as well as higher odds of 30-day re-admission when compared to other patients. Given the small number of events within this group of patients, it is possible that these results reflected a mere sampling bias due to skewed capturing of patients of these minor racial groups. Further research is needed to understand the impact of racial disparities on outcomes of patients belonging to racial groups other than White or Black.

Insurance status is known to significantly influence the outcomes of patients with venous thromboembolism as well as their risk for re-hospitalization following discharge [14–16]. In our study, uninsured patients had lower odds of undergoing surgical or catheter-directed embolectomy when compared to insured patients, while the odds of 30-day mortality or 30-day major bleeding were not significantly different. Our findings were similar to those of Farmakis et al. [14], who studied 1,124,204 hospitalizations for acute PE in the NIS and found that use of advanced therapies was lower in Medicaid beneficiaries (OR_adjusted_: 0.68) when compared to privately insured patients. Our results were also concordant with the results reported by Zumbrunn and colleagues [15], who studied 819 elderly patients in a Swiss prospective multicenter cohort and found that insurance status was *not* associated with mortality or risk of recurrent VTE. On the other hand, Wadhera and colleagues [16] investigated the long-term outcomes of 53,386 Medicare beneficiaries hospitalized for acute PE and found that socioeconomically disadvantaged patients had higher 1-year mortality rates as well as higher rates of 90-day re-admission. In our study, we only assessed the 30-day mortality and re-admission rates for patients and observed no significant difference between uninsured and insured patients; however, it remains unknown if their long-term outcomes were discrepant.

Limited English proficiency has been shown to influence the outcomes of patients hospitalized with acute medical conditions as well as the post-operative outcomes of patients undergoing elective surgery [44, 45]. In our study, the odds of 30-day mortality and 30-day major bleeding were not significantly different for patients who preferred a language other than English as compared to those who preferred English. At our institution, language interpreters via video conferencing or telephone are available at all times of the day and all days of the week to assist in caring for patients who are not proficient in English. Interestingly, odds of receiving surgical or catheter-directed embolectomy were higher for patients who preferred a language other than English as compared to those who preferred English. These findings suggest that the association of ethnicity with receipt of advanced therapies was not influenced by preferred language. However, it should be noted that we did not specifically record use of language interpreters as part of this research study. Additionally, a patient’s preference for a language other than English cannot be interpreted as signifying limited English proficiency. Therefore, further research is needed to explore the influence of limited English proficiency on outcomes of patients with acute PE managed by PERT.

The results of our study showed that PERT-based care may mitigate racial disparities in PE care and overall patient outcomes, although ethnic disparities still persisted in our patient cohort. While the results of our study are encouraging, there are a number of limitations that need to be borne in mind with regards to this study. Firstly, this study was performed at three urban teaching hospitals in New York City. Although patients treated at our hospitals were heterogeneous with respect to racial diversity and socioeconomic strata, we included only a small number of patients belonging to the Native Hawaiian, Pacific Islander and Alaskan Native racial groups. Therefore, it is unclear if the conclusions of our study are generalizable to those racial groups. Secondly, our study included a small proportion of patients of Hispanic or Latino ethnicity (*n* = 24, 8.3%) when compared to the larger group of patients of non-Hispanic/Latino ethnicity. It is unclear if this number (*n* = 24, 8.3%) was adequate to capture socioeconomic heterogeneity within the ethnic group of Hispanic or Latino patients. Thirdly, only a small number of patients included in our patient cohort experienced major bleeding at 30 days (*n* = 6), which could have underpowered our ability to detect subgroup differences. Nevertheless, the rate of 30-day major bleeding in our patient cohort was comparable to that reported by other investigators. Additionally, we could not account for lactate levels in our study as lactate levels were not uniformly checked in all patients; this could have potentially confounded the associations noted in our study. Moreover, we performed optimal full matching on a propensity score calculated from seven variables. It is possible that certain variables, which were not included in the propensity score, could have influenced the associations observed in our study. Lastly, we did not consider the economic status of the patients included in our study, which could have influenced the intra-racial variation in clinical outcomes.

## Conclusion

Within a cohort of acute PE patients managed by PERT, Black race was associated with higher odds of major bleeding and slightly lower odds of 30-day mortality. In contrast, Hispanic or Latino ethnicity was associated with lower odds of receiving CDT and catheter-directed or surgical embolectomy. Uninsured patients had lower odds of receiving systemic thrombolysis, CDT and catheter-directed or surgical embolectomy when compared to insured patients, although 30-day mortality and risk of major bleeding were not significantly different. These results suggest that PERT-based care somewhat mitigates racial disparities in the management of acute PE, although ethnic disparities in patient care still remain.

### Electronic supplementary material

Below is the link to the electronic supplementary material.

Supplementary Material 1

## Data Availability

The datasets generated and analyzed during the current study are available from the corresponding author on reasonable request.

## Abbreviations

BMI: Body mass index
BUN: Blood urea nitrogen
CI: Confidence interval
DOAC: Direct-acting oral anticoagulant
ESC: European Society of Cardiology
ICU: Intensive care unit
INR: International normalized ratio
IQR: Interquartile range
IRR: Incidence rate ratio
LOS: Length of stay
OR: Odds ratio
PE: Pulmonary embolism
PERT: Pulmonary embolism response team
PESI: Pulmonary embolism severity index
RDW: Red cell distribution width
RV: Right ventricle
VTE: Venous thromboembolism

## References

[1] Stein PD, Matta F (2011). Epidemiology and incidence: the scope of the problem and risk factors for development of venous thromboembolism. Crit Care Clin.

[2] Sedhom R, Megaly M, Elbadawi A, Elgendy IY, Witzke CF, Kalra S (2022). Contemporary National trends and outcomes of Pulmonary Embolism in the United States. Am J Cardiol.

[3] Stein PD, Matta F (2010). Acute pulmonary embolism. Curr Probl Cardiol.

[4] Salonia JS, Steiger D, Shapiro JM, Herzog E (2022). Pulmonary embolism response team: a Multidisciplinary Approach to Improve Pulmonary Embolism Management. Pulmonary embolism.

[5] Wright C, Goldenberg I, Schleede S, McNitt S, Gosev I, Elbadawi A (2021). Effect of a Multidisciplinary Pulmonary Embolism Response Team on Patient Mortality. Am J Cardiol.

[6] Chopard R, Campia U, Morin L, Jering KS, Almarzooq ZI, Snyder JE (2022). Trends in management and outcomes of pulmonary embolism with a multidisciplinary response team. J Thromb Thrombolysis.

[7] Konstantinides SV, Meyer G, Becattini C, Bueno H, Geersing GJ, Harjola VP et al. 2019 ESC guidelines for the diagnosis and management of acute pulmonary embolism developed in collaboration with the European Respiratory Society (ERS): the Task Force for the diagnosis and management of acute pulmonary embolism of the European Society of Cardiology (ESC). Eur Respir J. 2019;54(3). 10.1183/13993003.01647-2019 31473594

[8] Manly JJ (2006). Deconstructing race and ethnicity: implications for measurement of health outcomes. Med Care.

[9] Haider AH, Weygandt PL, Bentley JM, Monn MF, Rehman KA, Zarzaur BL (2013). Disparities in trauma care and outcomes in the United States: a systematic review and meta-analysis. J Trauma Acute Care Surg.

[10] Howell EA, Egorova NN, Janevic T, Brodman M, Balbierz A, Zeitlin J (2020). Race and ethnicity, Medical Insurance, and within-hospital severe maternal morbidity disparities. Obstet Gynecol.

[11] Kimball MM, Neal D, Waters MF, Hoh BL (2014). Race and income disparity in ischemic stroke care: nationwide inpatient sample database, 2002 to 2008. J Stroke Cerebrovasc Dis.

[12] Galiatsatos P, Sun J, Welsh J, Suffredini A (2019). Health disparities and Sepsis: a systematic review and Meta-analysis on the influence of race on Sepsis-related mortality. J Racial Ethn Health Disparities.

[13] Khanijahani A, Iezadi S, Gholipour K, Azami-Aghdash S, Naghibi D (2021). A systematic review of racial/ethnic and socioeconomic disparities in COVID-19. Int J Equity Health.

[14] Farmakis IT, Valerio L, Giannakoulas G, Hobohm L, Cushman M, Piazza G (2023). Social determinants of health in pulmonary embolism management and outcome in hospitals: insights from the United States nationwide inpatient sample. Res Pract Thromb Haemost.

[15] Zumbrunn B, Stalder O, Mean M, Limacher A, Tritschler T, Rodondi N (2019). Association between insurance status, anticoagulation quality, and clinical outcomes in patients with acute venous thromboembolism. Thromb Res.

[16] Wadhera RK, Secemsky EA, Wang Y, Yeh RW, Goldhaber SZ (2021). Association of Socioeconomic Disadvantage with Mortality and readmissions among older adults hospitalized for pulmonary embolism in the United States. J Am Heart Assoc.

[17] Bailey ZD, Krieger N, Agenor M, Graves J, Linos N, Bassett MT (2017). Structural racism and health inequities in the USA: evidence and interventions. Lancet.

[18] Holtgrave DR, Barranco MA, Tesoriero JM, Blog DS, Rosenberg ES (2020). Assessing racial and ethnic disparities using a COVID-19 outcomes continuum for New York State. Ann Epidemiol.

[19] Austin PC, Stuart EA (2017). Estimating the effect of treatment on binary outcomes using full matching on the propensity score. Stat Methods Med Res.

[20] Austin PC, Stuart EA (2015). Optimal full matching for survival outcomes: a method that merits more widespread use. Stat Med.

[21] Schultz J, Giordano N, Zheng H, Parry BA, Barnes GD, Heresi GA (2019). EXPRESS: a Multidisciplinary Pulmonary Embolism Response Team (PERT) - experience from a national multicenter consortium. Pulm Circ.

[22] Rosovsky R, Chang Y, Rosenfield K, Channick R, Jaff MR, Weinberg I (2019). Changes in treatment and outcomes after creation of a pulmonary embolism response team (PERT), a 10-year analysis. J Thromb Thrombolysis.

[23] Hussein EA, Semaan DB, Phillips AR, Andraska EA, Rivera-Lebron BN, Chaer RA et al. Pulmonary embolism response team for hospitalized patients with submassive and massive pulmonary embolism: A single-center experience. J Vasc Surg Venous Lymphat Disord. 2023;11(4):741-7 e2. 10.1016/j.jvsv.2023.03.002 PMC12433811 36906104

[24] Sathianathan S, Meili Z, Romero CM, Juarez JJ, Bashir R. Racial and gender disparities in the management of acute pulmonary embolism. J Vasc Surg Venous Lymphat Disord. 2024:101817. 10.1016/j.jvsv.2024.101817 PMC11523363 38296110

[25] Dronamraju VH, Lio KU, Badlani R, Cheng K, Rali P (2023). PERT era, race-based healthcare disparities in a large urban safety net hospital. Pulm Circ.

[26] Kucher N, Boekstegers P, Muller OJ, Kupatt C, Beyer-Westendorf J, Heitzer T (2014). Randomized, controlled trial of ultrasound-assisted catheter-directed thrombolysis for acute intermediate-risk pulmonary embolism. Circulation.

[27] Avgerinos ED, Jaber W, Lacomis J, Markel K, McDaniel M, Rivera-Lebron BN (2021). Randomized trial comparing standard Versus Ultrasound-assisted thrombolysis for Submassive Pulmonary Embolism: the SUNSET sPE Trial. JACC Cardiovasc Interv.

[28] Inci EK, Khandhar S, Toma C, Licitra G, Brown MJ, Herzig M (2023). Mechanical thrombectomy versus catheter directed thrombolysis in patients with pulmonary embolism: a multicenter experience. Catheter Cardiovasc Interv.

[29] Jaureguizar A, Ochoa Chaar CI, Muriel A, Vela Moreno JR, Weinberg I, Tufano A (2022). Comparison of full-dose vs moderate-dose systemic thrombolysis for the treatment of patients with Acute Pulmonary Embolism. Chest.

[30] Ismayl M, Ismayl A, Hamadi D, Aboeata A, Goldsweig AM (2024). Catheter-directed thrombolysis versus thrombectomy for submassive and massive pulmonary embolism: a systematic review and meta-analysis. Cardiovasc Revasc Med.

[31] Kuo WT, Banerjee A, Kim PS, DeMarco FJ, Levy JR, Facchini FR (2015). Pulmonary embolism response to Fragmentation, Embolectomy, and catheter thrombolysis (PERFECT): initial results from a prospective Multicenter Registry. Chest.

[32] Piazza G, Hohlfelder B, Jaff MR, Ouriel K, Engelhardt TC, Sterling KM (2015). A prospective, Single-Arm, Multicenter Trial of Ultrasound-Facilitated, Catheter-Directed, low-dose fibrinolysis for Acute massive and submassive pulmonary embolism: the SEATTLE II study. JACC Cardiovasc Interv.

[33] Tapson VF, Sterling K, Jones N, Elder M, Tripathy U, Brower J (2018). A Randomized Trial of the Optimum Duration of Acoustic Pulse Thrombolysis Procedure in Acute Intermediate-Risk Pulmonary Embolism: the OPTALYSE PE Trial. JACC Cardiovasc Interv.

[34] Toma C, Bunte MC, Cho KH, Jaber WA, Chambers J, Stegman B (2022). Percutaneous mechanical thrombectomy in a real-world pulmonary embolism population: interim results of the FLASH registry. Catheter Cardiovasc Interv.

[35] Semaan DB, Phillips AR, Reitz K, Sridharan N, Mulukutla S, Avgerinos E (2023). Improved long-term outcomes with catheter-directed therapies over medical management in patients with submassive pulmonary embolism-a retrospective matched cohort study. J Vasc Surg Venous Lymphat Disord.

[36] Giri J, Sista AK, Weinberg I, Kearon C, Kumbhani DJ, Desai ND (2019). Interventional therapies for Acute Pulmonary Embolism: current status and principles for the development of Novel evidence: a Scientific Statement from the American Heart Association. Circulation.

[37] Pruszczyk P, Klok FA, Kucher N, Roik M, Meneveau N, Sharp ASP (2022). Percutaneous treatment options for acute pulmonary embolism: a clinical consensus statement by the ESC Working Group on Pulmonary circulation and right ventricular function and the European Association of Percutaneous Cardiovascular Interventions. EuroIntervention.

[38] Gonsalves CF, Gibson CM, Stortecky S, Alvarez RA, Beam DM, Horowitz JM (2023). Randomized controlled trial of mechanical thrombectomy vs catheter-directed thrombolysis for acute hemodynamically stable pulmonary embolism: Rationale and design of the PEERLESS study. Am Heart J.

[39] Klok FA, Piazza G, Sharp ASP, Ni Ainle F, Jaff MR, Chauhan N (2022). Ultrasound-facilitated, catheter-directed thrombolysis vs anticoagulation alone for acute intermediate-high-risk pulmonary embolism: Rationale and design of the HI-PEITHO study. Am Heart J.

[40] Graham GN, Jones PG, Chan PS, Arnold SV, Krumholz HM, Spertus JA (2018). Racial disparities in patient characteristics and Survival after Acute myocardial infarction. JAMA Netw Open.

[41] Herd P, Mills MC, Dowd JB (2021). Reconstructing Sociogenomics Research: Dismantling Biological race and genetic essentialism narratives. J Health Soc Behav.

[42] Andreasen RO, Matthen M, Stephens C (2007). Biological conceptions of Race. Philosophy of Biology.

[43] Haider AH, Scott VK, Rehman KA, Velopulos C, Bentley JM, Cornwell EE (2013). Racial disparities in surgical care and outcomes in the United States: a comprehensive review of patient, provider, and systemic factors. J Am Coll Surg.

[44] Limaye NP, Matias WR, Rozansky H, Neville BA, Vise A, McEvoy DS (2024). Limited English proficiency and Sepsis mortality by race and ethnicity. JAMA Netw Open.

[45] Kunze KN, Estrada JA, Apostolakos J, Fu MC, Taylor SA, Gulotta LV (2023). Association between Limited English Language proficiency and disparities in length of Stay and Discharge Disposition after total shoulder arthroplasty: a retrospective cohort study. HSS J.

