# Supplementary material for: Association of race, ethnicity and insurance status with outcomes for patients with acute pulmonary embolism treated by PERT: a retrospective observational study
Source: Respir Res. 2024 Jun 24;25:259. doi: 10.1186/s12931-024-02872-5 (PMC11197331; doi:10.1186/s12931-024-02872-5)
Supplement: Supplementary file 1 — Supplementary Material 1 [file 12931_2024_2872_MOESM1_ESM.docx]

*Original Research*

**Association of race, ethnicity and insurance status with outcomes for patients with acute pulmonary embolism treated by PERT: a retrospective observational study**

Abdul Rehman^1^ (MBBS), Avinash Singh^2^ (MBBS), Priyanka Sridhar^3^ (MD), Hong Yu Wang^3^ (MD), Agostina Velo^3^ (MD), Destiny Nguyen^3^ (MD), Madeline Ehrlich^2^ (MPharm), Robert Lookstein^4^ (MD), David Steiger^2*^ (MD)

*^1^ Department of Medicine, Rutgers-New Jersey Medical School, Newark, New Jersey 07103, United States
^2^ Division of Pulmonary, Critical Care and Sleep Medicine, Department of Medicine, Icahn School of Medicine at Mount Sinai Health System, New York City, New York 10029, United States*

*^3^ Department of Medicine, Icahn School of Medicine at Mount Sinai Health System, New York City, New York 10029, United States*

*^4^ Department of Radiology, Icahn School of Medicine at Mount Sinai Health System, New York City, New York 10029, United States*

* Corresponding author

David J. Steiger, MD

Professor and Division Chief,

Mount Sinai Beth Israel,

Division of Pulmonary, Critical Care, and Sleep Medicine,

Department of Medicine,

Icahn School of Medicine at Mount Sinai,

New York City, NY 10019,
United States of America.

Tel: (212) 420-2377

david.steiger@mountsinai.org

**Supplementary Information**

This file contains 5 supplementary figures and 2 supplementary tables for the original research article “Association of race, ethnicity and insurance status with outcomes for patients with acute pulmonary embolism treated by PERT: a retrospective observational study” by Rehman and colleagues.


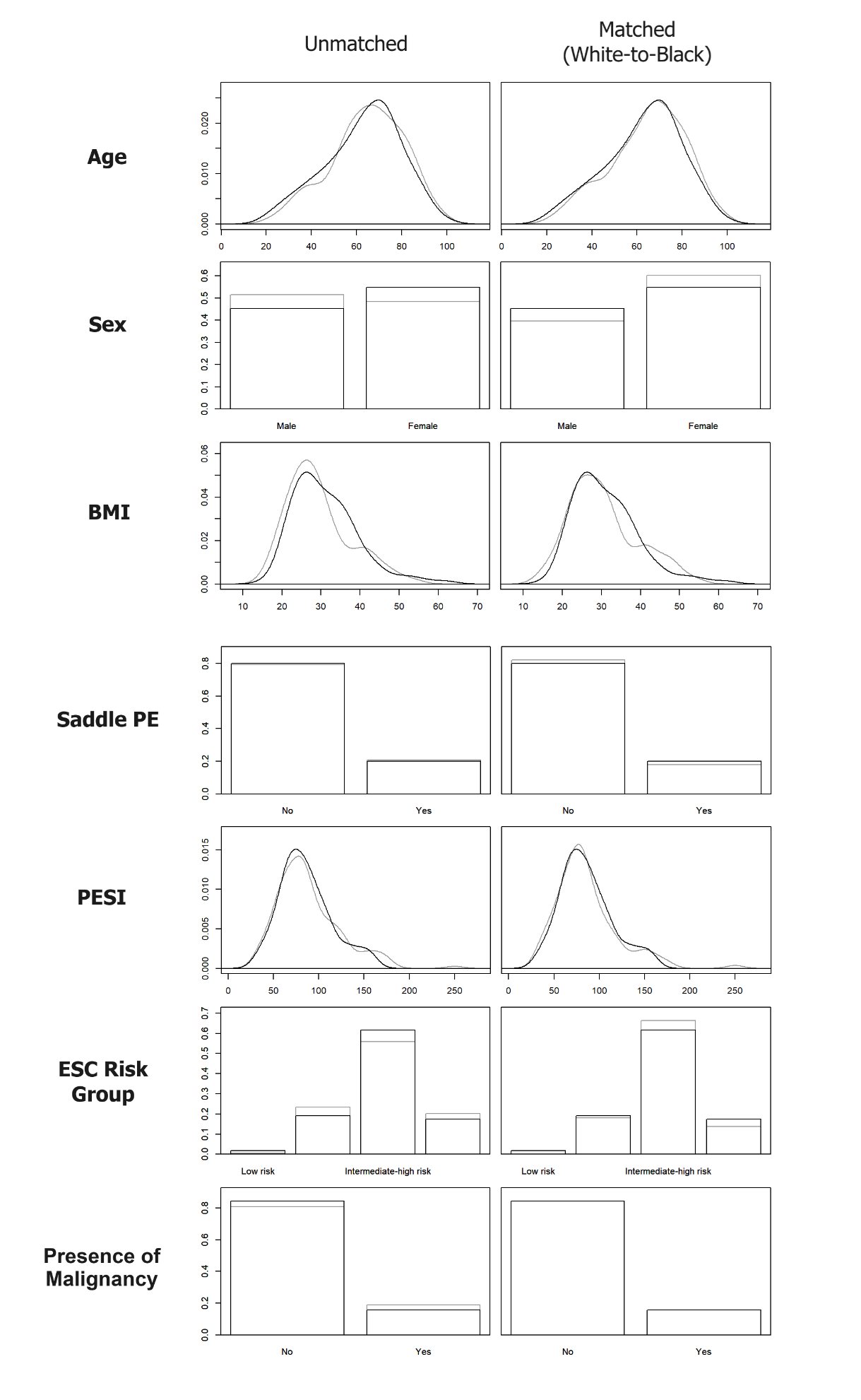


**Supplementary Figure 1:** **Density plot of the unmatched sample and matched sample (Black patients matched to White patients).** Propensity score weighting was performed using the optimal full matching specification in the *MatchIt* package in R. Propensity scores were calculated using generalized linear regression with a *probit* link function from the following variables: age, sex, body mass index, Pulmonary Embolism Severity Index (PESI) score, European Society of Cardiology (ESC) risk group, presence of a saddle pulmonary embolus and presence of malignancy. Black color represents Black patients, while gray color represents the control group.


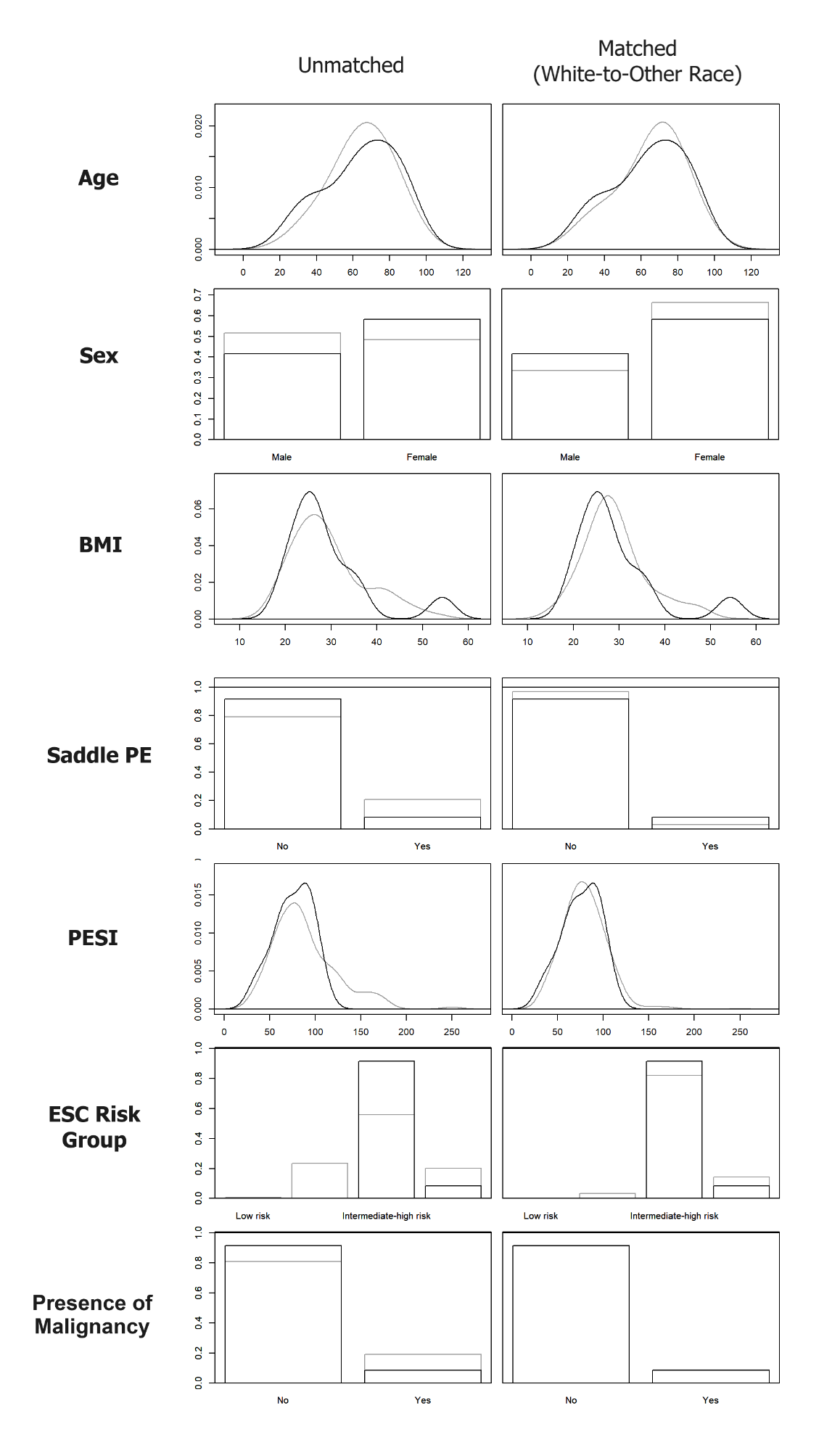


**Supplementary Figure 2:** **Density plot of the unmatched sample and matched sample (Patients of races other than White or Black matched to White patients).** Propensity score weighting was performed using the optimal full matching specification in the *MatchIt* package in R. Propensity scores were calculated using generalized linear regression with a *probit* link function from the following variables: age, sex, body mass index, Pulmonary Embolism Severity Index (PESI) score, European Society of Cardiology (ESC) risk group, presence of a saddle pulmonary embolus and presence of malignancy. Black color represents patients of races other than White or Black, while gray color represents the control group.


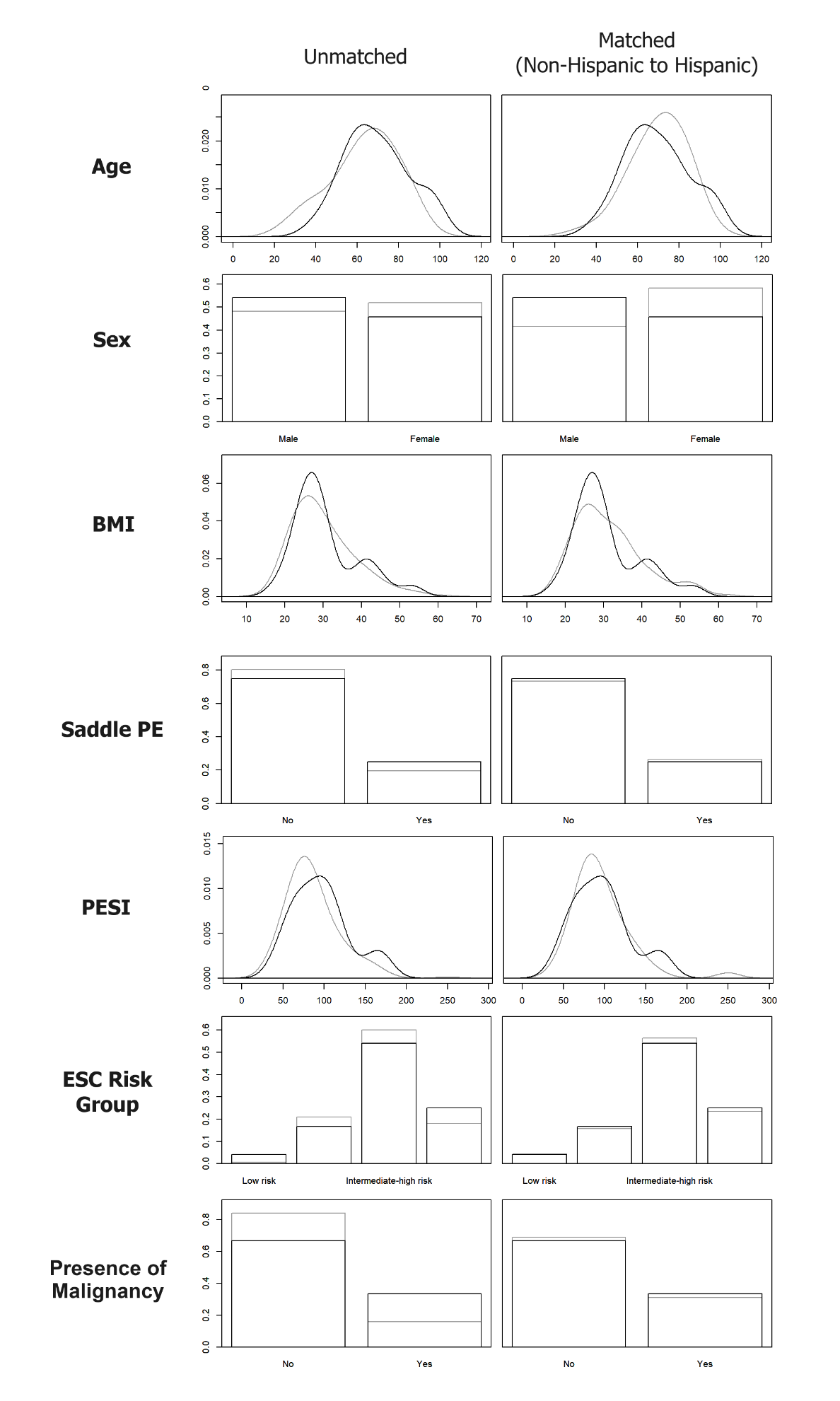


**Supplementary Figure 3:** **Density plot of the unmatched sample and matched sample (Patients of Hispanic or Latino ethnicity matched to patients of other ethnicities).** Propensity score weighting was performed using the optimal full matching specification in the *MatchIt* package in R. Propensity scores were calculated using generalized linear regression with a *probit* link function from the following variables: age, sex, body mass index, Pulmonary Embolism Severity Index (PESI) score, European Society of Cardiology (ESC) risk group, presence of a saddle pulmonary embolus and presence of malignancy. Black color represents Hispanic or Latino ethnicity patients, while gray color represents the control group.


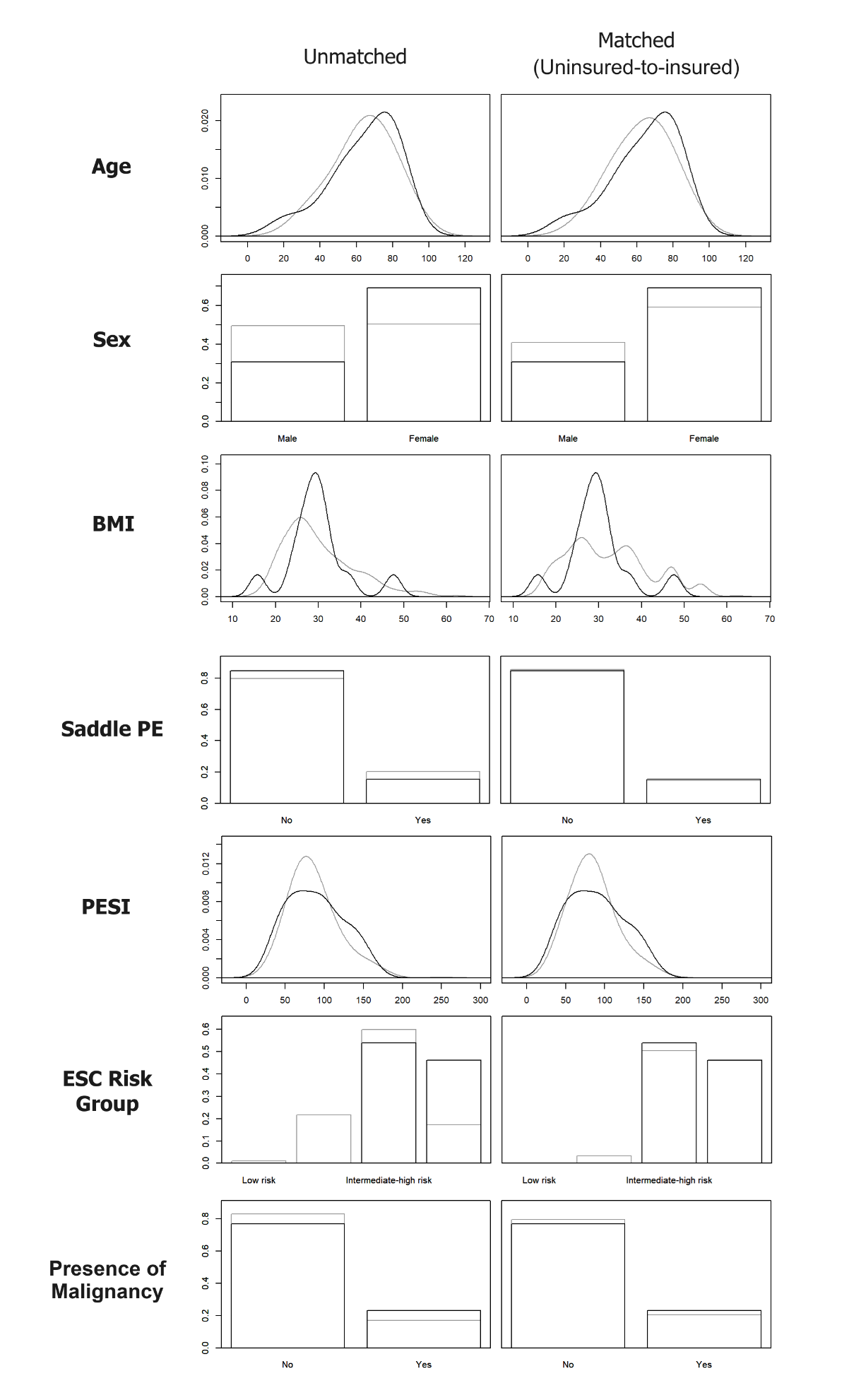


**Supplementary Figure 4:** **Density plot of the unmatched sample and matched sample (Uninsured patients matched to patients with insurance).** Propensity score weighting was performed using the optimal full matching specification in the *MatchIt* package in R. Propensity scores were calculated using generalized linear regression with a *probit* link function from the following variables: age, sex, body mass index, Pulmonary Embolism Severity Index (PESI) score, European Society of Cardiology (ESC) risk group, presence of a saddle pulmonary embolus and presence of malignancy. Black color represents uninsured patients, while gray color represents the control group.


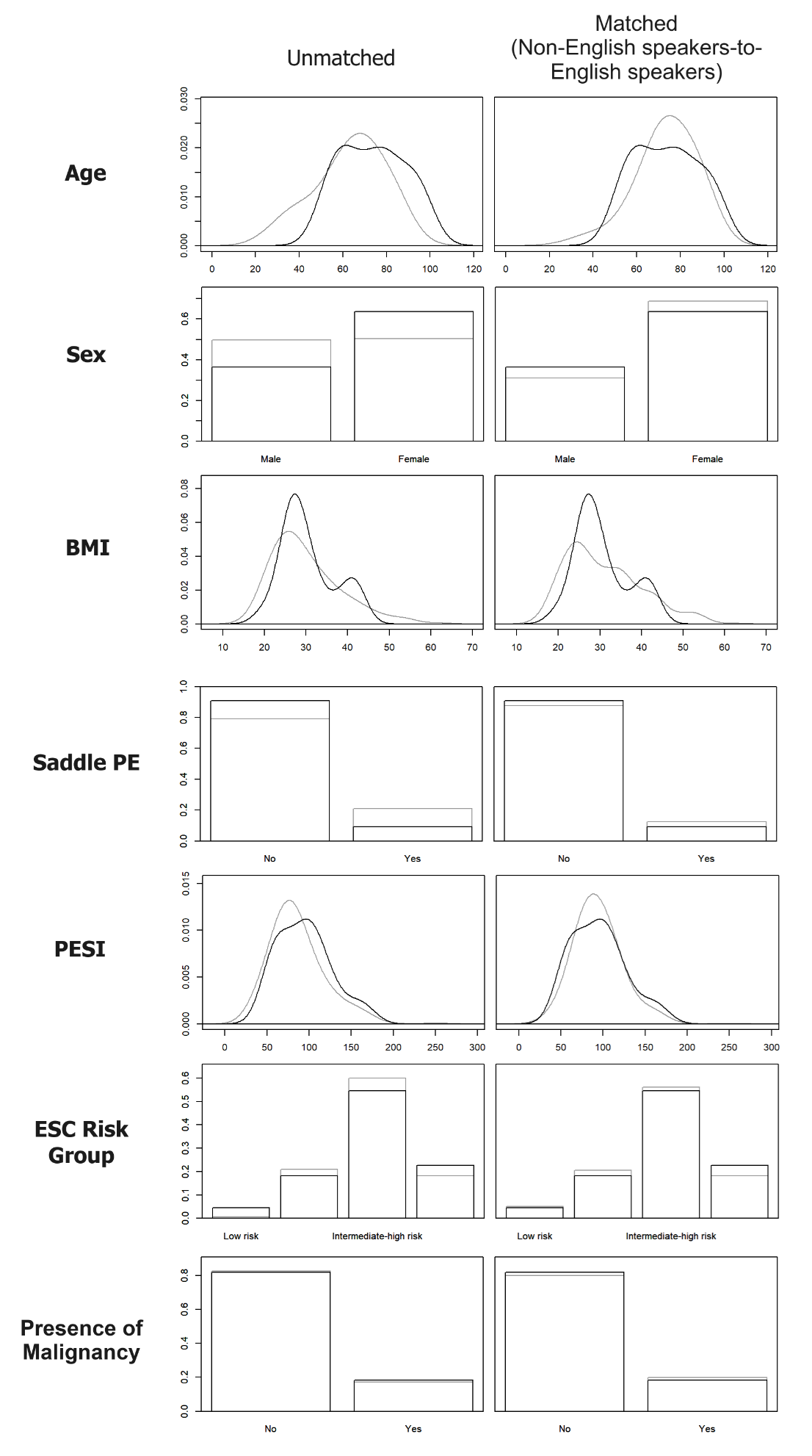


**Supplementary Figure 5:** **Density plot of the unmatched sample and matched sample (Patients who preferred a language other than English matched to patients who preferred English).** Propensity score weighting was performed using the optimal full matching specification in the *MatchIt* package in R. Propensity scores were calculated using generalized linear regression with a *probit* link function from the following variables: age, sex, body mass index, Pulmonary Embolism Severity Index (PESI) score, European Society of Cardiology (ESC) risk group, presence of a saddle pulmonary embolus and presence of malignancy. Black color represents patients who preferred a language other than English, while gray color represents the control group.
